# Memory association dynamics on neural network with dynamic synapses

**DOI:** 10.1186/1471-2202-15-S1-P128

**Published:** 2014-07-21

**Authors:** Yuichi Katori

**Affiliations:** 1 Institute of Industrial Science, The University of Tokyo, Japan

## 

Recent physiological studies revealed that the strength of the synaptic connections changes in a short period of time with short-term plasticity (STP) mechanism; these synapse is called dynamic synapse [1]. The synaptic strength decreases (depression synapse) or increases (facilitation synapse) with occurrence of the presynaptic spikes. The STP is suggested to contribute flexible information representation in the prefrontal cortex [2]. Dynamical properties of neural networks with STP have been intensively investigated [3]. In the associative memory network with STP, the STP contribute to generate variety of dynamical states including transitive dynamics among stored memory patterns.

In the present study, we further explore the dynamical properties of the associative memory network with stochastic binary neuron model. Changes in the synaptic transmission efficacy can be modeled with variables that represent the releasable neurotransmitter and the utilization parameter reflecting the calcium concentration on presynaptic terminal. We drive the dynamical mean field model that allows to analyze detailed bifurcation structure of network dynamics of the stochastic model. We evaluate the memory retrieve performance with applying external input.

## Conclusion

In the associative memory network with dynamic synapses, the network shows variety of dynamical state including memory retrieved states and transitive states among the memory patterns.

**Figure 1:**
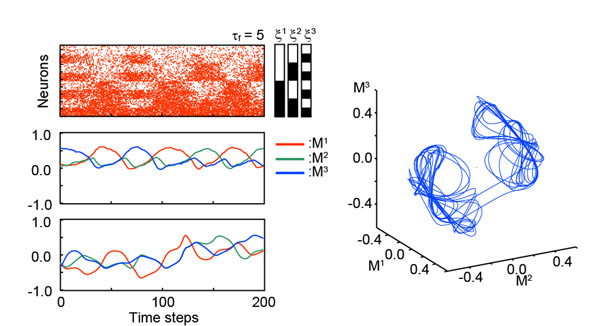
Responses on the associative memory network with dynamic synapses. Transitive dynamics on the stochastic model and the overlaps quantifying a similarity between the stored pattern and the state of the network.
